# Digital therapeutics for stress in health science students at under-resourced universities: A narrative review

**DOI:** 10.4102/hsag.v31i0.3301

**Published:** 2026-08-19

**Authors:** Bhekithemba Vellem, Nozuko G. Mangi

**Affiliations:** 1 Department of Nursing, Faculty of Medicine and Health Sciences, Walter Sisulu University, Mthatha, South Africa

**Keywords:** digital therapeutics, mobile health (mHealth), stress management, health science students, under-resourced universities, mental health

## Abstract

**Background:**

Health science students’ well-being and academic performance are important concerns in under-resourced universities. However, there is limited insight into mHealth interventions and institutional support for psychological stress driven by demanding curricula and socioeconomic disparities. Digital therapeutics (DTx) offer scalable technology-enabled support for stress management.

**Aim:**

This narrative review explores how DTx can complement or enhance existing stress management approaches for health science students in under-resourced South African universities.

**Methods:**

A narrative literature search was conducted across four databases: PubMed, Scopus, Web of Science and Google Scholar. Publications were from 2020 to 2024 on DTx addressing stress among university students, in low-resource settings and theoretical foundations. PRISMA guidelines informed reporting, and thematic synthesis followed Braun and Clarke. Theoretical frameworks informed analysis of effectiveness, the digital divide and mental health service integration.

**Results:**

The synthesis of nine studies, institutional reports and grey literature yielded four main themes: infrastructural and financial constraints, psychological and sociocultural barriers, facilitators and success factors and integration with university mental health services.

**Conclusion:**

Barriers included poor connectivity, high data costs, limited digital literacy, stigma around mental health and weak institutional support. Effective DTx adoption in low-resource settings requires overcoming barriers and strengthening policy, practice and research.

**Contribution:**

Digital therapeutics have the potential to play an important role in addressing mental health inequities among health science students in South Africa and similar contexts. The study contributes insights into digital health interventions to enhance support systems in higher education.

## Introduction

The phenomenon of stress among health science students has garnered significant global attention because of rising levels of psychological distress reported within this group (Almansoof et al. 2024). Health science education is inherently demanding, involving high academic workloads, compulsory work-integrated learning (WIL) placements in clinical settings and rigorous assessment requirements, all of which contribute significantly to student stress (Santiago et al. 2024). These stressors are intensified by the competitive nature of academic advancement and the ethical responsibilities demanded by health professions, resulting in a persistent psychological burden on students (Bin Abdulrahman, Alghamdi & Abahussain 2025). Worldwide, this has led to an increase in anxiety, depression, burnout and other mental health issues reported by health science students (Agyapong-Opoku et al. 2023; Almansoof et al. 2024). Additionally, the overlap of academic pressures with personal and social difficulties further worsens stress, increasing susceptibility to adverse outcomes such as poor academic performance and declining well-being (Khatake et al. 2022). Students in health sciences at South African universities face a complex range of challenges that heighten their risk of psychological stress (Henderson et al. 2021). This stress is compounded by ongoing socioeconomic inequalities in South Africa, which raise external stressors like financial insecurity, limited healthcare access and insufficient institutional support (Chiramba & Ndofirepi 2023; Plagerson & Mthembu 2019). The situation could be more pronounced at under resource settings. The Coronavirus Disease 2019 (COVID-19) pandemic has exacerbated these issues by disrupting traditional learning, promoting social isolation and creating uncertainty about academic progression and career prospects (Fichardt, Van Vuuren & Van der Merwe 2023). The cumulative impact of academic stressors and structural inequalities underscores the urgent need for alternative and supplementary stress management strategies, particularly in South African universities. Although many of these universities provide free student support services, counselling and academic guidance, these services are often overburdened and may be insufficient to meet growing demand, especially in resource-constrained settings (Bantjes et al. 2023). Notably, digital therapeutics (DTx) and mobile health (mHealth) interventions have demonstrated significant potential as scalable and accessible solutions to address the complex mental health needs of students in resource-constrained settings (Choudhury et al. 2023; Bidargaddi et al. 2020; Hunt et al. 2023; Oliveira et al. 2021). In South Africa, mHealth platforms have gained traction as part of the broader digital health strategy, offering Internet-based cognitive behavioural therapy (CBT), self-help tools and teletherapy services that can bypass traditional barriers to care (Botes 2025; Discovery Health 2025; National Department of Health 2020). However, their implementation in limited resource setting contexts requires overcoming infrastructural challenges, including the digital divide and unreliable connectivity, to ensure equitable access and effectiveness (Li, Zhang & Chen 2023). This context presents a compelling rationale for investigating how DTx can effectively alleviate stress among this vulnerable population by leveraging South Africa’s widespread mobile phone penetration. As the COVID-19 pandemic heightened awareness of the mental health crisis, it simultaneously accelerated technological adoption, spotlighting digital health tools as crucial components in the mental health ecosystem. Addressing student stress in under-resourced universities is not only timely but necessary, given the insufficiency of existing mental health resources, the pervasive effects of socioeconomic inequities and the digital opportunity spaces that have emerged.

### Literature review

Digital therapeutics are evidence-based, regulated software interventions designed to prevent, manage or treat medical conditions, validated through randomised controlled trials and subject to regulatory oversight (Jiang et al. 2025; Kim et al. 2025). Examples include Sleepio for insomnia, reSET for substance use disorder and BlueStar for diabetes. In contrast, mobile health (mHealth) refers to a broader category of mobile-based applications that support health promotion, education and service delivery, such as SMS reminders, teletherapy platforms and wellness apps (Li et al. 2025). Mobile technology encompasses the hardware and infrastructure, including smartphones, tablets, wearables and Internet connectivity, that enable both DTx and mHealth to function (Alhassan, Ozturkcan & Cavdar 2026; Alkhuzaimi et al. 2025). This distinction is crucial in under-resourced university contexts, where DTx provide clinical-grade efficacy but are often costly, while mHealth tools offer more accessible, sometimes free support with variable validation. Costs of DTx vary considerably depending on the condition and delivery model. Subscription fees typically range from US$10 per month to US$120 annually (Grand View Research 2023; Kim et al. 2025). Beyond licensing, students in South Africa face significant barriers to mobile data use, with affordability a major concern (Musekiwa, Lubinga & Masiya 2025). Infrastructure and training costs further add to implementation expenses, though DTx can reduce overall healthcare costs by 30% – 40% compared to in-person care (Lee et al. 2025). These financial considerations highlight the challenge of integrating DTx into under-resourced universities without subsidies or institutional support. Globally, DTx applications have demonstrated efficacy across diverse conditions. Sleepio in the UK and US have shown effectiveness for insomnia; SilverCloud Health in Europe has supported depression and anxiety management. The Kaia Health in Germany has addressed musculoskeletal pain, and Wellthy Therapeutics in India has targeted diabetes and cardiovascular disease (Gerke et al. 2020; Yan, Balijepalli & Druyts 2021). In low- and middle-income countries, Peru’s digital self-care service reduced depression, anxiety and stress among university populations (Yslado-Méndez et al. 2025), demonstrating the feasibility of digital interventions in resource-constrained settings. Free and low-cost stress management applications have also been developed for university students. The Roadmap App incorporates positive psychology exercises and has reduced depression and anxiety (Jayaraj et al. 2025). Cerina provides CBT-based support for anxiety, while the ANTI-DA App uses gamification to address depression and anxiety (Gopalan et al. 2025; Stanford & Just 2025). In South Africa, the Panda app offers free peer support and guided sessions, and MomConnect demonstrates scalable mHealth delivery models (Barron et al. 2018; Fedhealth 2024). These examples illustrate that while DTx remain limited in accessibility, mHealth tools provide more immediate and cost-effective avenues for stress management in under-resourced universities. For health science students in South Africa, the integration of DTx into university mental health frameworks remains underexplored. Digital therapeutics offer clinical-grade efficacy but are often restricted to insured populations, whereas mHealth tools provide broader accessibility. A blended approach that combines open-source or free mHealth platforms with selective DTx, where feasible, may maximise reach and equity in under-resourced university contexts (Acharya 2022; Kim et al. 2024). This underscores the importance of investigating digital therapeutic interventions to alleviate stress among health science students in disadvantaged settings. South African university students, especially those in health science faculties at under-resourced institutions, face a unique constellation of stressors (Henderson et al. 2021; Lesunyane, Ramano & Van Niekerk 2025). The country’s legacy of inequality manifests in disparities across education, healthcare and social support systems, creating environments that intensify stress for students from disadvantaged backgrounds. Many institutions lack adequate infrastructure and personnel to support mental health, leaving students to manage academic demands, personal adversity and social isolation with limited institutional assistance (Alotaibi et al. 2025; Julius et al. 2024). Beyond academic rigour, students contend with financial strain, restricted access to mental health services and insufficient psychosocial support. These external pressures exacerbate stress and contribute to poor mental health outcomes, with a high prevalence of depression, anxiety and trauma-related symptoms, particularly among marginalised groups (Pillay & Kriel 2021). Social determinants such as housing insecurity, safety concerns and limited access to essential services further compound these challenges. The digital divide, marked by unequal access to the Internet, devices and digital literacy, adds another layer of complexity, especially as digital mental health solutions gain traction. The widespread use of mobile technology among South African youth offers a promising avenue for mental health interventions in under-resourced university settings. Mobile phone penetration is high even among economically disadvantaged populations, providing a scalable, cost-effective platform for delivering support. Mobile health (mHealth) technologies, which utilise mobile devices to disseminate health information and interventions, can bypass common barriers such as geographic isolation, shortage of mental health professionals and stigma around help-seeking (Wang et al. 2021). Despite this promise, the digital divide remains a significant obstacle. Disparities in Internet access, device ownership and digital literacy must be addressed through interventions that are contextually sensitive and adaptable to infrastructural and cultural constraints (Liu, Wu & Tang 2025; Ndibalema 2025). Evidence from other low- and middle-income countries supports the efficacy of mHealth platforms in improving mental health literacy, resilience and coping strategies (Madonsela et al. 2023). These tools offer flexible, private and accessible support mechanisms that can be particularly beneficial in environments where traditional services are limited or stigmatised. Traditional university-based interventions such as counselling, workshops and peer support groups have shown effectiveness but face deployment challenges in under-resourced settings. These include a lack of qualified professionals, logistical barriers and academic pressures that limit student participation. Moreover, existing services may not be culturally attuned to the specific stress dynamics faced by South African health science students, many of whom navigate intersecting socioeconomic hardships (Musakuro & Gie 2025). The expansion of mobile phone use presents an opportunity to bridge these gaps. However, the potential of mHealth remains underexplored in South African universities, where digital literacy gaps and infrastructural limitations require tailored strategies. The success of digital health tools depends on their cultural relevance, accessibility and alignment with user needs, factors often overlooked in solutions developed in high-income contexts (Borghouts et al. 2021). There is a critical gap in understanding how DTx can be effectively integrated into existing university mental health frameworks or serve as alternatives where such services are absent. This gap encompasses not only technological feasibility but also psychological and sociocultural dimensions that influence student engagement. Recent work on South African universities highlights that while e-health tools are being explored, adoption remains constrained by infrastructural limitations, readiness and cultural factors (Musakuro & Gie 2024). Addressing these factors is essential to developing interventions that are both effective and equitable in under-resourced South African university settings. This research gap encompasses not only the technological and infrastructural feasibility but also the psychological and sociocultural dimensions affecting student engagement with digital interventions. In particular, how DTx might integrate with and enhance existing university mental health services, or provide alternatives where such services are absent, is insufficiently understood. Most DTx in South Africa are subscription based or linked to healthcare providers, which creates a cost barrier in under-resourced university settings. For example, Discovery Health’s SilverCloud iCBT requires membership (Discovery Health 2023), while platforms like TheraGen and Neeuro are commercial offerings (Ortiz 2026). Locally developed apps such as Vimbo (Steyn & Slabbert 2023) and Kuamsha (Moffett et al. 2025) have demonstrated strong engagement in trials but are mainly available through research programmes or partnerships. Free or subsidised options exist: Wysa is provided at the University of the Western Cape (2023), and global apps like Headspace and Woebot are accessible but often require subscriptions for full features (Hoffman et al. 2023; Militello et al. 2022). While DTx can enhance university mental health services, its integration in under-resourced contexts depends on affordability, institutional support and equitable access (Mudau, Jithoo & Dietrich 2024). Addressing this gap requires a focused investigation into the potential utility, barriers and facilitators associated with digital therapeutic applications in these settings (Borghouts et al. 2021). Against this backdrop, this narrative review sought to answer the following key research question:

*How can digital therapeutic interventions support or improve existing mental health services within universities, especially when traditional services are limited, inconsistent or inaccessible among health science students in South Africa?*

This question is positioned within the broader imperative to develop scalable, culturally sensitive and cost-effective stress management strategies that address the unique challenges faced by this vulnerable student population. The purpose of this narrative review was to explore how DTx can complement or enhance existing stress management approaches for health science students in under-resourced South African universities. The objective of this narrative review was to explore how DTx can complement or enhance existing stress management approaches in health science.

### Theoretical framework

The narrative review employed two complementary theoretical frameworks as lenses to guide inquiry and interpret findings: Lazarus and Folkman’s Transactional Model of Stress and Coping (TMSC) (Lazarus & Folkman 1984) and the Technology Acceptance Model (TAM) (Davis 1989). The deployment of these theoretical frameworks was essential in framing the review as they provide a structured lens through which to conceptualise the complex phenomena of stress and technology adoption. Given the multifactorial nature of student stress and the innovative character of DTx, theories ground the investigation by delineating key constructs, guiding intervention design and offering explanatory models for behavioural and psychosocial outcomes. The interplay between psychological models of stress and coping with theories explaining technology acceptance provides a multidimensional perspective necessary to holistically address the research question. *Transactional Model of Stress and Coping:* This model conceptualises stress as a transactional dynamic where individuals appraise environmental demands relative to available coping resources. Primary appraisal evaluates whether stressors are perceived as threats or challenges, while secondary appraisal assesses coping options. Coping strategies are broadly problem focused (altering the stressor) or emotion focused (managing emotional responses). This framework elucidates how health science students experience and negotiate academic and socio-environmental stressors and guides targeting cognitive appraisals and coping skill enhancement in digital interventions. *Technology Acceptance Model:* TAM posits that perceived usefulness and perceived ease of use determine users’ intention to adopt technology. These perceptions shape attitudes and actual usage behaviour. In the context of mHealth for under-resourced students, TAM explicates how factors like interface design, cultural relevance, digital literacy, privacy concerns and infrastructural stability influence acceptance. Incorporating TAM aids in designing user-centric interventions and training that bolster adoption and sustainability. *Integrating TMSC and TAM:* These models together offer a multidimensional view by examining the psychological processes behind stress management and the behavioural factors influencing technology adoption. This integration helps in assessing both the effectiveness of intervention content and the practicality of its delivery methods. It further emphasises the need for culturally and contextually appropriate, user-friendly platforms that can foster meaningful engagement and lasting impact within under-resourced settings.

## Research methods and design

### Design

This study adopted an integrative narrative review approach to synthesise and analyse diverse bodies of literature on student mental health, DTx and mobile health interventions in under-resourced university contexts. An integrative approach allows for the inclusion of empirical, theoretical and contextual evidence to develop a comprehensive understanding of the feasibility and applicability of digital mental health interventions in South African higher education settings. An integrative review is a rigorous yet flexible literature review methodology that synthesises diverse bodies of evidence, including qualitative, quantitative and mixed-methods research, to develop a comprehensive understanding of complex phenomena and to generate new knowledge frameworks (Zhou & Yin 2025). This approach serves as a critical methodological tool for researchers seeking to synthesise large, heterogeneous bodies of literature that span multiple disciplines and study designs. This integrative narrative review explores and summarises the literature on DTx and related mHealth interventions for stress management among health science students at under-resourced universities, particularly in South Africa. The methodology combined structured electronic searches with a thematic synthesis to integrate quantitative and qualitative evidence as well as theoretical frameworks relevant to both stress and technology adoption. The review followed the Preferred Reporting Items for Systematic Reviews and Meta-Analyses (PRISMA) 2018 guidelines to ensure methodological rigour and transparent reporting. The methodology combined comprehensive database searches with thematic synthesis to integrate findings. Theoretical frameworks relevant to stress and technology adoption, namely the TMSC by (Lazarus & Folkman 1984) and the TAM (Davis 1989), were used to guide interpretation and thematic organisation.

### Search strategy

A literature search was conducted across four major databases: PubMed, Scopus, Web of Science and Google Scholar. A manual supplementary search was also conducted using Google Scholar and SciSpace to identify additional relevant sources and ensure comprehensive coverage, particularly of grey literature. The search encompassed publications from 01 January 2020 to 31 December 2024, including both peer-reviewed articles and grey literature. The rationale behind choosing the five recent periods was to capture the most recent evidence on DTx, a rapidly evolving field. This period also highlights the increased adoption of digital health interventions during and following the COVID-19 pandemic, which notably affected student stress and the utilisation of digital solutions in higher education. Boolean search operators were developed for each database using a combination of controlled vocabulary (Medical Subject Headings [MeSH] for PubMed) and free-text keywords. Search terms included: (“digital therapeutics” OR “digital therapy” OR “mobile health” OR “mHealth” OR “eHealth” OR “digital mental health” OR “internet-based therapy” OR “mobile applications” AND “stress” OR “psychological stress” OR “stress management” OR “mental health” OR “anxiety” OR “depression” OR “burnout” AND “health science students” OR “medical students” OR “nursing students” OR “allied health students” OR “health professions students” OR “university students” AND “South Africa” OR “sub-Saharan Africa” OR “low-resource settings” OR “resource-limited settings” OR “developing countries” OR “low and middle income countries” OR “LMICs”.

### Inclusion criteria

Studies were included if they met the following criteria:

- Reported on digital health, mHealth or digital mental health interventions.
- Focused on university students, preferably health science students or analogous population (higher education, low- and middle-income settings).
- Included studies from South Africa or sub-Saharan Africa OR relevant evidence from other low-resource contexts.
- Provided empirical findings or reviews on effectiveness, barriers and facilitators or user engagement.
- Published in English between 2020 and 2024 to capture the recent relevant 5-year studies, as digital and mobile health studies are fast and emerging.

### Exclusion criteria

- Studies not reporting digital health or mHealth outcomes.
- Studies only describing traditional in-person interventions or unrelated populations (e.g. primary school children).
- Publications in languages other than English.

### Study selection process

**FIGURE 1:**
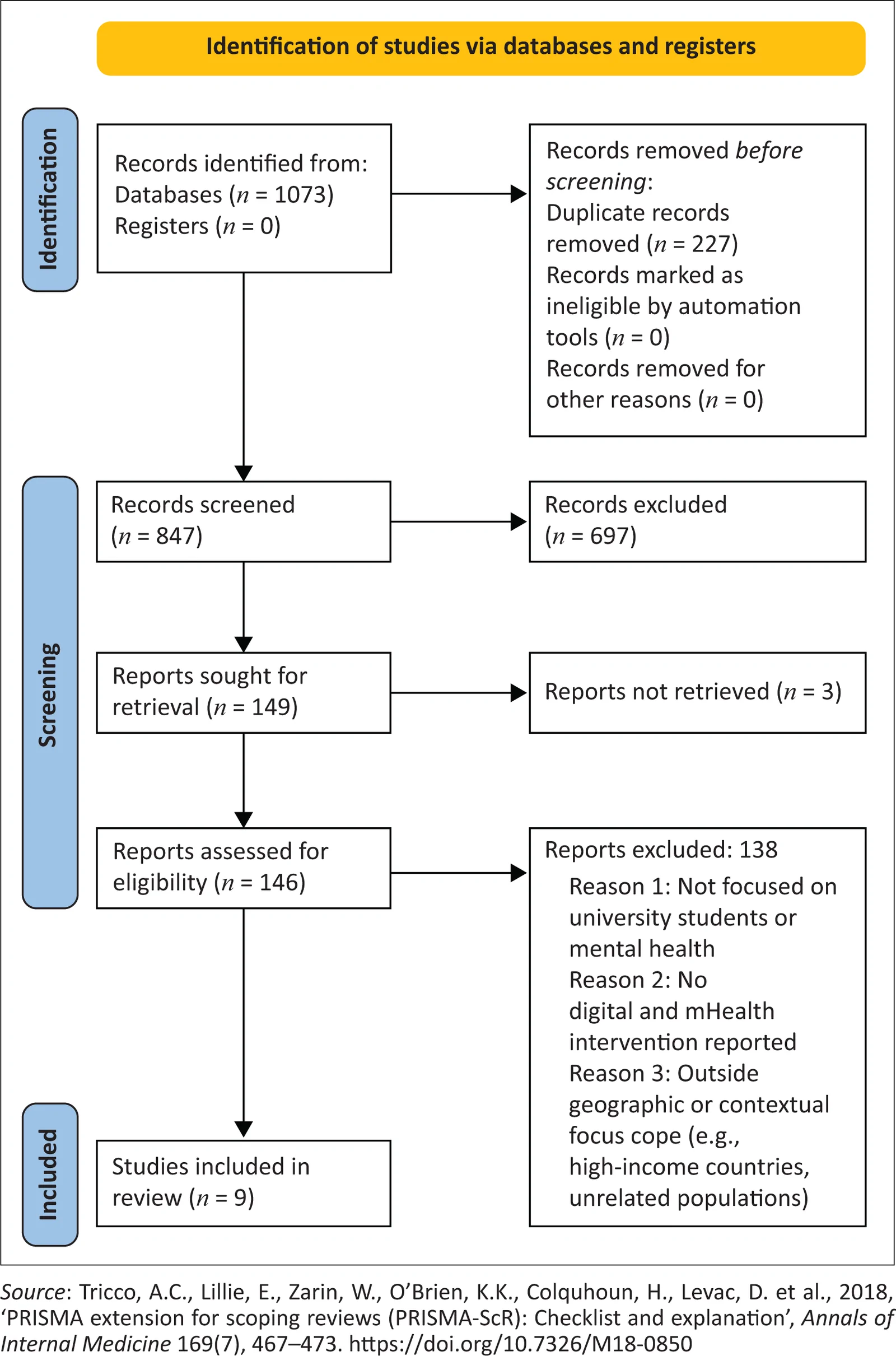
Preferred Reporting Items for Systematic Reviews and Meta-Analyses 2018 flow diagram for new systematic reviews, which included searches of databases and registers only.

The study selection followed PRISMA guidelines (Tricco et al. 2018). A total of 1073 records were identified through database searches. After removing 227 duplicates, 847 records remained for title and abstract screening, of which 697 were excluded because they did not meet the predefined inclusion criteria. The remaining 149 reports were sought for retrieval; three could not be retrieved. Consequently, 146 full-text articles were assessed for eligibility. Of these, 138 were excluded because of irrelevance to digital mental health interventions, non-student populations or lack of stress-related outcomes. Nine studies met the inclusion criteria and were included in the review. The authors (B.V and N.G.M) independently screened titles and abstracts (*n* = 149), followed by full-text assessment (*n* = 146) for eligibility. Disagreements were resolved through discussion and consensus, maintaining an unbiased study selection among the two reviewers. The progression of studies through the screening stages was mapped in a PRISMA flow diagram, further clarifying inclusion and exclusion decisions. Figure 1 illustrates the PRISMA 2018 flow diagram used to present the identification, screening, eligibility and inclusion of records in the review.

### Data extraction and synthesis

In each study included in this review, data were extracted by both reviewers. The extracted information covered the author, year of publication and study location; the study design and methodological approach; and any theoretical frameworks used. Details about the characteristics of the study population or sample were recorded, along with the type and mode of digital intervention applied. Key findings were documented, including evidence of intervention effectiveness and barriers and facilitators to implementation. Additionally, technological and cultural insights relevant to the adoption and uptake of the interventions were noted. The thematic synthesis in this review was guided by Braun and Clarke’s (2022) reflexive thematic analysis (RTA). It was used to identify patterns in effectiveness, the digital divide, barriers and facilitators and integration with existing mental health services. The process involved six stages: (1) familiarisation with the data through repeated reading of included studies; (2) systematic coding using both deductive (guided by the review aims: effectiveness, digital divide, barriers and facilitators and integration) and inductive approaches to identify emerging insights; (3) generating initial themes by grouping related codes; (4) iterative review and refinement of themes for coherence and relevance; (5) defining and naming the themes and (6) producing the final narrative synthesis.

### Ethical considerations

Ethical approval was granted by the Walter Sisulu University Faculty of Medicine and Health Sciences Health Research Committee with ethics number: 247/2024.

## Results

Nine studies met the inclusion criteria and were included in this narrative review. The studies were published between 2022 and 2024 and comprised three mixed-methods studies, two cross-sectional surveys, two qualitative and narrative synthesis studies (one qualitative and one narrative review), one institutional implementation report and one randomised controlled trial protocol. All studies were conducted in South African contexts, with five focusing specifically on university student populations, three addressing broader digital mental health implementation relevant to higher education settings and one focusing on young people in rural South Africa. The results reviewed across the studies included feasibility, acceptability, usability, perceived usefulness, accessibility and contextual barriers and facilitators to implementation. Thematic analysis was guided by Braun and Clarke (2022). Reflexive Thematic Analysis methodology identified four main themes: infrastructural and financial constraints, psychological and sociocultural barriers, facilitators and success factors and integration with university mental health services. The included studies are summarised in a Table 1, which was reviewed by two independent reviewers (B.V and N.G.M).

**TABLE 1:** Summary of included studies.

No	Author(s)	Year	Title / setting	Methodology	Digital platform used	Key findings
1.	Bröcker et al.	2024	Counsellor-supported PTSD coach intervention in a resource-constrained South African setting	Mixed-methods feasibility trial	PTSD coach mobile app	Demonstrated feasibility, acceptability and improved coping outcomes; infrastructural barriers noted.
2.	Mudau et al.	2024	Demand, practicality, and acceptability of a mental-health application for South African university students	Cross-sectional and mixed-methods	Custom mental health mobile app	High student interest but barriers included data costs, privacy and digital literacy.
3.	Hunt et al.	2023	South African university students’ experiences of online group CBT	Qualitative study	Online group CBT via video-conferencing platform	Online GCBT improved stress coping; cultural tailoring enhanced acceptability.
4.	ISRCTN Trial 47089643 (UCT)	2023–ongoing	Randomised controlled trial of online GCBT for South African university students	RCT protocol	Online CBT platform (university hosted)	Ongoing trial; illustrates growing local evidence base for structured online therapies.
5.	Gbollie et al.	2023	Student attitudes towards digital mental health solutions	Cross-sectional survey	Survey of multiple apps and platforms	Perceived usefulness and ease of use strongly predicted adoption.
6.	University of the Western Cape (Wysa evaluation)	2023	Implementation of Wysa AI-enabled mental health app at UWC	Institutional report	Wysa AI chatbot app	High engagement observed; institutional support and cultural adaptation critical to uptake.
7.	Mitchell, Gradidge and Ntlokwana	2024	Hybrid models of digital and face-to-face counselling in South African universities	Mixed-methods	Hybrid digital counselling platforms + face-to-face sessions	Hybrid approaches improved accessibility; institutional readiness remains a challenge.
8.	Mbunge et al.	2022	Virtual healthcare and digital technologies in South Africa (COVID-19 context)	Systematic review	Various virtual healthcare platforms	Adoption of digital mental health tools increasing; barriers include infrastructure and data costs.
9.	Mindu et al.	2023	Digital mental health interventions for young people in rural South Africa: Prospects and challenges for implementation	Narrative review and qualitative synthesis	Various digital mental health apps and platforms (mHealth/eHealth)	Highlighted infrastructural and affordability barriers (data costs, device access), cultural acceptability issues and recommended targeted measures such as subsidised data packages, campus-wide Wi-Fi and low-bandwidth/offline-compatible applications.

Note: Please see full reference list of this article: Vellem, B. & Mangi, N.G., 2026, ‘Digital therapeutics for stress in health science students at under-resourced universities: A narrative review’, *Health SA Gesondheid* 31(0), a3301. https://doi.org/10.4102/hsag.v31i0.3301 for more information.

CBT, cognitive behavioural therapy; PTSD, Post-traumatic stress disorder; GCBT, Group cognitive behavioural therapy; RCT, Randomised controlled trial; AI, Artificial intelligence.

### Thematic results

#### Theme 1: Infrastructural and financial constraints

A consistent finding across the reviewed studies was the significant impact of infrastructural and financial limitations on the implementation and uptake of digital mental health interventions. Studies by Mudau et al. (2024), Mbunge et al. (2022) and the University of the Western Cape Wysa implementation report (2023) highlighted unreliable Internet connectivity, high data costs and limited access to suitable devices as major barriers, particularly for students in under-resourced and rural university settings. Bröcker et al. (2024) similarly reported infrastructural challenges that affect sustained engagement with counsellor-supported digital interventions. Under-resourced universities, particularly those located in rural provinces, face significant challenges, including unreliable Internet connectivity, intermittent electricity supply and limited access to smartphones or compatible devices (Hunt et al. 2023; Mindu et al. 2023). Financial constraints intertwine with infrastructural deficits. A significant proportion of students are unable to afford requisite data plans or upgrade devices capable of running digital therapeutic applications consistently (Mindu et al. 2023). The high costs of mobile data usage create barriers to continuous, reliable engagement, thereby reducing the potential impact of these interventions. To mitigate these challenges, studies including Mudau et al. (2024), Mbunge et al. (2022), Bröcker et al. (2024), the UWC Wysa evaluation (2023) and Mindu et al. (2023) have recommended targeted measures such as subsidised data packages, institutional investment in campus-wide Wi-Fi and the development of low-bandwidth or offline-compatible applications for resource-poor settings. Failure to address these foundational barriers risks reinforcing exclusionary patterns instead of promoting equitable access to mental health support.

#### Theme 2: Psychological and sociocultural barriers

Beyond the tangible infrastructural limitations, a complex array of psychological and sociocultural factors also impedes digital mental health intervention uptake. While stigma surrounding mental illness remains a barrier in many South African contexts (Mbunge et al. 2022; Mudau et al. 2024), several studies also reported that the private and confidential nature of DTx may reduce stigma and increase willingness to seek support. For example, Gbollie et al. (2023) found that perceived anonymity and confidentiality positively influenced students’ attitudes towards digital mental health solutions. Cultural narratives and historical interactions with healthcare systems further influence attitudes towards DTx. In various contexts, psychological distress may be interpreted through spiritual or traditional lenses, rendering Western-style digital interventions less acceptable or perceived as foreign (Mindu et al. 2023). These dynamics shape students’ primary appraisal of digital tools, often leading to avoidance or reliance on emotion-focused coping strategies instead of engagement. Hunt et al. (2023) demonstrated that cultural tailoring of online group CBT substantially enhanced acceptability among South African university students, illustrating the importance of stakeholder engagement. To overcome these barriers, this review highlights the importance of user-centred design approaches. Co-creation with students enables the development of interventions that are linguistically, culturally and contextually relevant, thereby enhancing trust, acceptability and usability (Mindu et al. 2023). Additionally, robust privacy safeguards embedded within digital platforms are critical to fostering confidence in their security and ethical handling of sensitive information.

#### Theme 3: Facilitators and success factors

Facilitators of successful adoption included perceived usefulness, ease of use and institutional endorsement. Gbollie et al. (2023) demonstrated that these factors were strong predictors of students’ intention to use digital mental health tools. The UWC Wysa evaluation (2023) further illustrated that institutional support, cultural adaptation and integration into existing student wellness services significantly enhanced engagement. Mitchell et al. (2024) highlighted the effectiveness of hybrid models combining digital and face-to-face counselling in improving accessibility while maintaining clinical oversight. Participatory design processes represent a central facilitator. Hunt et al. (2023) demonstrated that cultural tailoring enhanced acceptability, illustrating the importance of stakeholder engagement. Gbollie et al. (2023) found that perceived usefulness and ease of use strongly predicted adoption, suggesting that user-centred approaches yield measurable benefits for technology acceptance. Institutional support plays a decisive role. The University of the Western Cape’s evaluation of the Wysa AI-enabled mental health app (2023) documented high engagement and emphasised that institutional support and cultural adaptation were critical to uptake. Similarly, Mitchell et al. (2024) reported that hybrid models of digital and face-to-face counselling improved accessibility, though they noted that institutional readiness remains a challenge. Peer support networks integrated within digital platforms have proven effective in enhancing user engagement. The ISRCTN Trial 47089643 (2023–ongoing) is building the evidence base for structured online therapies, illustrating the field’s growing momentum. Multisector collaborations involving universities, governmental bodies, non-governmental organisations and technology developers facilitate scalability and sustainability. Training programs aimed at improving digital literacy among both university staff and students are critical enablers that enhance platform navigation skills, confidence and sustained use of interventions. Collectively, these success factors increase students’ coping resources, favouring effective problem-focused strategies and resilience building. Collectively, these success factors increase students’ coping resources, favouring effective problem-focused strategies and resilience building.

#### Theme 4: Integration with university mental health services

The successful integration of DTx into existing university mental health frameworks has become a crucial factor in their overall effectiveness and sustainability. The ISRCTN Trial 47089643 protocol (2023), ongoing, illustrates increasing institutional investment in structured, evidence-based online interventions for university students. Similarly, Mitchell et al. (2024) reported that hybrid care models improved access to support while allowing for stepped referral to in-person services when needed. This model is supported by broader evidence showing that digital platforms can complement rather than replace traditional services. These models effectively balance accessibility, personalisation and clinical oversight.

However, many under-resourced universities face significant gaps in infrastructure, trained personnel and organisational readiness. Mitchell et al. (2024) explicitly noted that institutional readiness remains a challenge, a finding consistent with the broader barriers identified in other studies. The University of the Western Cape’s Wysa evaluation (2023) highlighted that institutional support and cultural adaptation are critical to successful integration. The establishment and enforcement of ethical institutional policies governing informed consent, data management and appropriate platform usage are foundational to cultivating student trust and engagement. Capacity-building initiatives to improve digital literacy and self-efficacy among users and providers further strengthen platform acceptance and effective use.

## Discussion

This narrative review synthesised evidence on the potential of DTx and mobile health (mHealth) interventions to support stress management among health science students at under-resourced South African universities. The findings, guided by Braun and Clarke (2022) RTA, the nine included studies reveal distinct patterns of barriers, facilitators and implementation challenges. Drawing on the TMSC (Lazarus & Folkman 1984) and the TAM (Davis 1989), this section interprets the results through theoretical and contextual lenses.

### Theme 1: Infrastructural and financial barriers

Infrastructural limitations emerged as a consistent barrier across multiple included studies. Mudau et al. (2024) identified data costs and digital literacy as critical obstacles to the uptake of a university mental health application, despite high student interest. Similarly, Bröcker et al. (2024) documented infrastructural barriers in their feasibility trial of a PTSD intervention, while Mbunge et al. (2022) found, in their systematic review, that barriers to virtual healthcare adoption in South Africa included infrastructure deficits and data costs. These findings align with the TMSC, which posits that environmental stressors such as unreliable connectivity and unaffordable data reduce students’ capacity for problem-focused coping by restricting access to digital support tools. The TAM further illuminates how infrastructural barriers diminish perceived ease of use, thereby reducing the likelihood of technology adoption. The nine included studies collectively underscore that without systemic investment in digital infrastructure and subsidised data access, DTx risk exacerbating rather than alleviating existing inequities. Addressing these challenges requires systemic interventions, including investment in digital infrastructure, subsidisation of data and devices and the development of low-bandwidth, offline-compatible applications. Without such foundational support, DTx risk reinforcing existing inequities rather than mitigating them.

### Theme 2: Psychological and sociocultural barriers

Beyond tangible infrastructural constraints, psychological and sociocultural factors significantly shaped student engagement with digital mental health tools. Mudau et al. (2024) reported that privacy concerns were a substantial barrier, while Hunt et al. (2023) demonstrated that cultural tailoring of online group CBT substantially enhanced acceptability among South African university students. The UWC Wysa AI-enabled mental health app evaluation revealed that cultural adaptation was critical to user uptake. These findings demonstrate that digital tools perceived as misaligned with students’ cultural values or mental health beliefs face resistance to adoption. The TAM explains this pattern: cultural narratives and historical healthcare experiences shape students’ attitudes towards technology. Gbollie et al. (2023) provided direct empirical evidence that perceived usefulness and ease of use strongly predicted adoption intention, suggesting that interventions addressing cultural relevance and user-centred design are essential for overcoming sociocultural barriers. The included studies collectively emphasise that co-creation with students and robust privacy safeguards are foundational to bridging technological innovation with cultural realities. To overcome these barriers, interventions must be culturally sensitive, linguistically appropriate and designed with robust privacy safeguards. User-centred design approaches that involve students in co-creation processes are essential for enhancing trust, relevance and engagement. The included studies collectively emphasise that co-creation with students and robust privacy safeguards are foundational to bridging technological innovation with cultural realities.

### Theme 3: Facilitators and success factors

Despite barriers, several facilitators emerged from the included studies that promoted effective implementation of DTx. Hunt et al. (2023) demonstrated that cultural tailoring of online CBT improved stress coping and acceptability, while the Wysa evaluation at UWC highlighted that institutional support and cultural adaptation were critical success factors. Mitchell et al. (2024) found that hybrid models combining digital and face-to-face counselling improved accessibility in South African universities. Gbollie et al. (2023) revealed that perceived usefulness and ease of use were strong predictors of adoption, indicating that user-centred design directly supports engagement. From a transactional model perspective, these facilitators enhance students’ coping resources by providing accessible, relevant support mechanisms. The TAM complements this understanding: when digital tools are perceived as useful and easy to use, behavioural intention to engage increases substantially. Collectively, the nine studies demonstrate that participatory design, institutional commitment and cultural adaptation are critical enablers for sustainable implementation.

### Theme 4: Integration with university mental health services

The successful integration of DTx into existing university mental health frameworks emerged as essential for effectiveness and sustainability. Mitchell et al. (2024) documented that hybrid care models combining digital platforms with face-to-face counselling improved accessibility, though institutional readiness remained a challenge. The study by Bröcker et al. (2024) demonstrated the feasibility and improved coping outcomes in their mixed-methods trial, establishing proof of concept for integrated digital interventions. The ongoing ISRCTN randomised controlled trial illustrates the growing local evidence base for structured online therapies within South African university contexts. However, institutional barriers to integration were evident. The Wysa evaluation demonstrated that while engagement was high, successful implementation required institutional support and policy alignment. The transactional model underscores the importance of tiered coping mechanisms, where digital tools provide low-intensity support and escalate complex cases to human providers. The TAM emphasises that institutional trust and organisational readiness shape both attitudes and actual usage behaviour. The included studies collectively demonstrate that ethical implementation encompassing clear policies on informed consent, data governance and privacy protection is foundational to cultivating sustained student engagement and institutional legitimacy. Hybrid care models that combine digital platforms with face-to-face counselling enable stepwise interventions tailored to students’ needs. However, many under-resourced institutions lack the infrastructure, trained personnel and organisational readiness to support such integration. Ethical implementation requires clear policies on informed consent, data governance and privacy. These findings further highlight the need for capacity-building initiatives to improve digital literacy among students, thereby enhancing engagement and self-efficacy. Sustained engagement requires participatory design, institutional commitment, integration of peer support and multisector collaboration to effectively enhance students’ coping resources, fostering resilience and promoting problem-focused stress management.

### Limitations and future research

This review is constrained by methodological heterogeneity, with most evidence drawn from feasibility, qualitative and cross-sectional studies rather than large-scale RCTs, limiting conclusions about clinical efficacy. The focus on university student populations reduces generalisability to broader youth groups, and findings from relatively well-resourced institutions may not apply to rural or severely under-resourced settings. Barriers such as data costs and infrastructural deficits were noted across several studies but rarely quantified, and most research assessed short-term feasibility without long-term follow-up, leaving sustained engagement unclear. For policymakers, this review highlights the need to invest in digital infrastructure, subsidised access and regulatory frameworks that ensure safe, equitable and culturally responsive deployment of mental health technologies. The clinicians and university support services should integrate these tools into hybrid care models, supported by staff training and student digital literacy initiatives. Future research must move beyond exploratory designs to include rigorous evaluations, such as randomised controlled trials and implementation studies, while also examining intersectional factors, such as gender, disability and rurality, that shape digital engagement and access equity. These steps are essential to ensure that digital mental health solutions are not only scalable but also inclusive and sustainable (Gbollie et al. 2023).

## Conclusion

This narrative review set out to investigate how digital therapeutic interventions can support or enhance mental health services for health science students in under-resourced South African universities. Guided by Braun and Clarke (2022) methodology, the TMSC of Lazarus and Folkman (1984) and the TAM by Davis (1989), the review synthesised evidence from nine studies. Interventions such as mobile apps, online CBT and AI-enabled chatbots were feasible and acceptable, especially when culturally tailored and institutionally supported, with adoption driven by perceived usefulness, ease of use and hybrid care models. The comprehensive review of current literature indicates that DTx delivered through mobile platforms can effectively reduce stress and improve mental health outcomes among health science students, particularly in resource-constrained university settings. However, persistent infrastructural, psychological and equity-related barriers must be addressed to ensure sustainable and inclusive implementation. Ultimately, the review affirms the potential of DTx and mHealth interventions to transform student mental health support in low-resource settings while calling for policy reform, clinical integration and rigorous, context-specific research to guide future practice.
